# 4-{2-[3-(2-Ammonioacetamido)propanamido]ethyl}-1*H*-imidazol-3-ium dichloride

**DOI:** 10.1107/S1600536808035952

**Published:** 2008-11-29

**Authors:** Katalin Selmeczi, Bernard Henry, Emmanuel Wenger, Slimane Dahaoui

**Affiliations:** a Groupe Complexation et Cinétique en Milieu Microhétérogène, Laboratoire SRSMC (UMR 7565 CNRS - Université Henri Poincaré Nancy 1), Nancy Université, BP 70239, F-54506 Vandoeuvre-lès-Nancy Cedex, France; b Laboratoire de Cristallographie et de Modélisation des Matériaux, Minéraux et Biologiques LCM3B (UMR 7036 CNRS - Université Henri Poincaré, Nancy 1), Nancy Université, BP 70239, F-54506 Vandoeuvre-lès-Nancy Cedex, France

## Abstract

Mol­ecules of the title compound, Gly-β-Ala-Histamine dihydro­chloride, C_10_H_19_N_5_O_2_^2+^·2Cl^−^, are linked by N—H⋯O and N—H⋯Cl hydrogen bonds into two-dimensional polymeric sheets parallel to the (011) plane, forming a stacked structure along the *a* axis. The parallel layers are also inter­linked alternately by different N—H⋯Cl hydrogen bonds, forming a three-dimensional framework.

## Related literature

For the complexation abilities of oligopeptides towards different metals, see: Kozlowski *et al.* (1999 ▶); Gajda *et al.* (1996 ▶). For bond lengths and angles in other oligopeptides, see: Itoh *et al.* (1977 ▶). For hydrogen-bond motifs, see: Bernstein *et al.* (1995 ▶). For related literature, see: Henry *et al.* (1993 ▶).
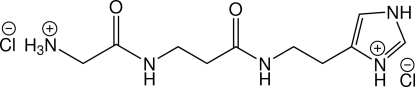


## Experimental

### 

#### Crystal data

C_10_H_19_N_5_O_2_^2+^·2Cl^−^
*M* *_r_* = 312.20
Triclinic, 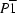

*a* = 7.2923 (10) Å
*b* = 8.2215 (11) Å
*c* = 13.0767 (15) Å
α = 81.702 (11)°
β = 77.863 (11)°
γ = 69.543 (12)°
*V* = 715.98 (16) Å^3^
*Z* = 2
Mo *K*α radiation
μ = 0.46 mm^−1^
*T* = 110 (2) K
0.30 × 0.20 × 0.12 mm

#### Data collection

Oxford Diffraction Xcalibur-Saphire2 CCD diffractometer
Absorption correction: numerical (*ABSORB*; DeTitta, 1985 ▶) *T*_min_ = 0.874, *T*_max_ = 0.952
12727 measured reflections
3307 independent reflections
1798 reflections with *I* > 2σ(*I*)
*R*_int_ = 0.061

#### Refinement

*R*[*F* ^2^ > 2σ(*F* ^2^)] = 0.046
*wR*(*F* ^2^) = 0.111
*S* = 0.97
3307 reflections
172 parameters
H-atom parameters constrained
Δρ_max_ = 0.51 e Å^−3^
Δρ_min_ = −0.35 e Å^−3^

### 

Data collection: CryslisCCD (Oxford Diffraction, 2003 ▶); cell refinement: *CrysAlis RED* (Oxford Diffraction, 2003 ▶); data reduction: *CrysAlis RED*; program(s) used to solve structure: *SHELXS97* (Sheldrick, 2008 ▶); program(s) used to refine structure: *SHELXL97* (Sheldrick, 2008 ▶); molecular graphics: *ORTEP-3 for Windows* (Farrugia, 1997 ▶); software used to prepare material for publication: *enCIFer* (Allen *et al.*, 2004 ▶).

## Supplementary Material

Crystal structure: contains datablocks global, I. DOI: 10.1107/S1600536808035952/cs2096sup1.cif

Structure factors: contains datablocks I. DOI: 10.1107/S1600536808035952/cs2096Isup2.hkl

Additional supplementary materials:  crystallographic information; 3D view; checkCIF report

## Figures and Tables

**Table 1:** Hydrogen-bond geometry (Å, °)

*D*—H⋯*A*	*D*—H	H⋯*A*	*D*⋯*A*	*D*—H⋯*A*
N1—H1′⋯Cl1	0.88	2.27	3.083 (4)	153
N2—H2′⋯O1^i^	0.88	1.81	2.670 (4)	165
N3—H3′⋯O2^i^	0.88	2.07	2.927 (4)	165
N4—H4′⋯Cl1^ii^	0.88	2.31	3.192 (4)	178
N5—H5C’⋯Cl2^iii^	0.91	2.32	3.152 (4)	152
N5—H5*B*′⋯Cl2	0.91	2.32	3.191 (4)	160
N5—H5*A*′⋯Cl1^iv^	0.91	2.31	3.161 (4)	156

Symmetry codes: (i) 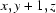; (ii) 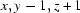; (iii) 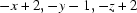; (iv) 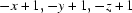.

**Fig. 1.**
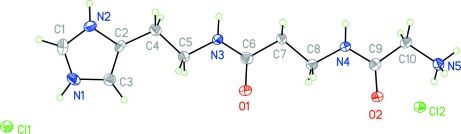
The molecular structure of the title salt with the atom-numbering scheme. Displacement ellipsoids are drawn at the 50% probability level.

**Fig. 2.**
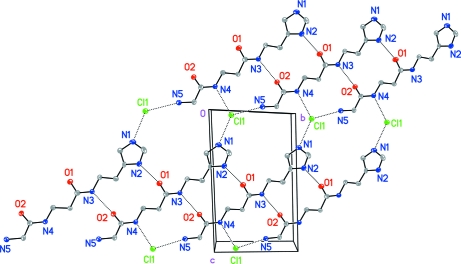
A partial packing diagram of (I), viewed along the a axis, showing the N—H···O and N—H···Cl1 hydrogen bonds. H atoms have been omitted for clarity.

**Fig. 3.**
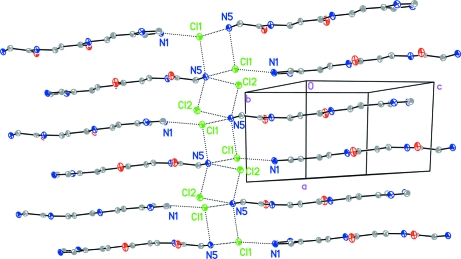
A view of the crystal packing of (I), showing the alternation of N5—H···Cl1 and N5—H···Cl2 hydrogen bonds (dashed lines) between two layers along the a axis. H atoms have been omitted for clarity.

## supplementary crystallographic information

### Comment

Serum albumin (SA) is the most abundant protein in human, and considered as the trace metal carrier between tissues and blood. To mimicking the coordination site in SA many oligopeptides have been synthesized and their complexation abilities towards different metals (Cu, Ni, Co, Mn, etc.) have been studied (Kozlowski *et al.*, 1999, Gajda *et al.*, 1996). We report here the molecular structure of the pseudo-tripeptide Glycyl-β-Alanyl-Histamine dihydrochloride (I) as a potential model compound which was synthesized in two steps from histamine hydrochloride, BOC-β-Alanine (BOC: *N*-(*tert*-butoxycarbonyl)) and BOC-Glycine. The asymmetric unit consists of the bicationic form of the pseudo-tripeptide and two chloride anions (Fig.1). The organic cation is essentially planar (maximum deviation from the mean plane is 0.102 (4) Å). The bond distances and angles of the peptide bonds and the protonated imidazolium rings are close to the values measured for other oligopeptides (Itoh *et al.*, 1977). Ions in the title salt are interlinked by two types of hydrogen bridges in the crystal. The N2 and N3 nitrogen atoms form strong N—H···O hydrogen bonds with O1^i^ and O2^i^ carbonyl oxygen atoms of neighbouring pseudo-tripeptide molecules, respectively [symmetry codes: (i) *x*, *y* + 1, *z*], giving an *R*_2_^2^(14) hydrogen-bonded ring motif (Bernstein *et al.*, 1995). The N1, N4 and N5 nitrogen atoms form N—H···Cl1 hydrogen bonds with Cl1, Cl1^ii^ and Cl1^iv^, respectively [symmetry codes: (ii) *x*, *y* - 1, *z* + 1 and (iv) -*x* + 1, -*y* + 1, -*z* + 1], and are engaged in two other cyclic patterns (*R*_2_^3^(13) and *R*_3_^5^(22)). This complex hydrogen bond framework gives a two-dimensional polymer parallel to the (011) plane (Fig.2). Layers are linked along the *a* axis and Cl1 and Cl2 atoms are alternatively involved. The distances between the two layers are 2.914 (4) Å and 3.747 (4) Å (N5···Cl1···N5 and N5···Cl2···N5, respectively) (Fig. 3).

### Experimental

The title compound was synthesized in two steps. First, β-Ala-histamine was prepared from *N*-(*tert*-butoxycarbonyl)-β-alanine and histamine dihydrochloride according to the procedure described earlier (Henry *et al.*, 1993). Using the same method in the second step, the title compound was obtained from the reaction of *N*-(*tert*-butoxycarbonyl)-glycine on β-Ala-histamine. Suitable crystals were obtained by slow diffusion of ethyl acetate into the methanolic solution of the title compound.

### Refinement

All H atoms bonded to C and N atoms were initially located from difference Fourier maps. Nevertheless, all the H atoms were constrained in a riding motion approximation with fixed bond lengths and *U*_iso_ parameters: C_aryl_–H = 0.95 Å, with *U*_iso_(H) = 1.2*U*_eq_(C); C_methylene_–H = 0.99 Å, with *U*_iso_(H) = 1.2*U*_eq_(C); N–H = 0.88 Å, with *U*_iso_(H) = 1.2*U*_eq_(N); N_amine_–H = 0.91 Å, with *U*_iso_(H) = 1.5*U*_eq_(N).

### Crystal data

**Table:**

C_10_H_19_N_5_O_2_^2+^·2Cl^–^	*Z* = 2
*M**_r_* = 312.20	*F*_000_ = 328
Triclinic, *P*1	*D*_x_ = 1.448 Mg m^−^^3^
Hall symbol: -P 1	Mo *K*α radiation λ = 0.71073 Å
*a* = 7.2923 (10) Å	Cell parameters from 12727 reflections
*b* = 8.2215 (11) Å	θ = 2.7–29.2º
*c* = 13.0767 (15) Å	µ = 0.46 mm^−^^1^
α = 81.702 (11)º	*T* = 110 (2) K
β = 77.863 (11)º	Prism, colourless
γ = 69.543 (12)º	0.30 × 0.20 × 0.12 mm
*V* = 715.98 (16) Å^3^	

### Data collection

**Table:**

Oxford Diffraction Xcalibur-Saphire2 CCD diffractometer	3307 independent reflections
Radiation source: fine-focus sealed tube	1798 reflections with *I* > 2σ(*I*)
Monochromator: graphite	*R*_int_ = 0.061
*T* = 110(2) K	θ_max_ = 29.2º
ω scan	θ_min_ = 2.7º
Absorption correction: numerical(ABSORB; DeTitta, 1985)	*h* = −9→9
*T*_min_ = 0.874, *T*_max_ = 0.952	*k* = −11→11
12727 measured reflections	*l* = −17→16

### Refinement

**Table:**

Refinement on *F*^2^	Secondary atom site location: difference Fourier map
Least-squares matrix: full	Hydrogen site location: inferred from neighbouring sites
*R*[*F*^2^ > 2σ(*F*^2^)] = 0.046	H-atom parameters constrained
*wR*(*F*^2^) = 0.111	*w* = 1/[σ^2^(*F*_o_^2^) + (0.0489*P*)^2^] where *P* = (*F*_o_^2^ + 2*F*_c_^2^)/3
*S* = 0.97	(Δ/σ)_max_ < 0.001
3307 reflections	Δρ_max_ = 0.51 e Å^−^^3^
172 parameters	Δρ_min_ = −0.35 e Å^−^^3^
Primary atom site location: structure-invariant direct methods	Extinction correction: none

### Fractional atomic coordinates and isotropic or equivalent isotropic displacement parameters (Å^2^)

**Table:**

	*x*	*y*	*z*	*U*_iso_*/*U*_eq_	
O1	0.7290 (4)	0.3622 (3)	0.54649 (15)	0.0302 (6)	
O2	0.7180 (3)	−0.1826 (2)	0.77310 (14)	0.0255 (5)	
N1	0.7705 (4)	1.0791 (3)	0.25403 (18)	0.0255 (6)	
H1'	0.7720	1.0903	0.1859	0.031*	
N2	0.7640 (4)	1.1387 (3)	0.40854 (18)	0.0230 (6)	
H2'	0.7614	1.1961	0.4609	0.028*	
N3	0.7314 (4)	0.5944 (3)	0.61436 (17)	0.0217 (6)	
H3'	0.7289	0.6443	0.6701	0.026*	
N4	0.7164 (4)	0.0643 (3)	0.83121 (17)	0.0205 (6)	
H4'	0.7173	0.1161	0.8854	0.025*	
N5	0.7507 (4)	−0.3688 (3)	0.95958 (18)	0.0223 (6)	
H5C'	0.7717	−0.4211	1.0243	0.033*	
H5B'	0.8438	−0.4329	0.9097	0.033*	
H5A'	0.6275	−0.3610	0.9505	0.033*	
C1	0.7671 (5)	1.2013 (4)	0.3094 (2)	0.0271 (7)	
H1	0.7668	1.3152	0.2829	0.033*	
C2	0.7655 (4)	0.9685 (3)	0.4167 (2)	0.0189 (7)	
C3	0.7717 (5)	0.9304 (4)	0.3183 (2)	0.0222 (7)	
H3	0.7759	0.8228	0.2977	0.027*	
C4	0.7614 (5)	0.8637 (4)	0.5201 (2)	0.0217 (7)	
H4B	0.6470	0.9288	0.5709	0.026*	
H4A	0.8842	0.8465	0.5473	0.026*	
C5	0.7448 (5)	0.6879 (3)	0.5113 (2)	0.0201 (7)	
H5B	0.6251	0.7041	0.4814	0.024*	
H5A	0.8626	0.6195	0.4638	0.024*	
C6	0.7229 (4)	0.4335 (4)	0.6247 (2)	0.0217 (7)	
C7	0.7099 (5)	0.3428 (3)	0.7336 (2)	0.0195 (7)	
H7B	0.5899	0.4119	0.7796	0.023*	
H7A	0.8276	0.3333	0.7634	0.023*	
C8	0.7003 (5)	0.1626 (3)	0.7299 (2)	0.0203 (7)	
H8B	0.5730	0.1730	0.7097	0.024*	
H8A	0.8098	0.0998	0.6761	0.024*	
C9	0.7297 (4)	−0.1029 (4)	0.8434 (2)	0.0190 (7)	
C10	0.7655 (5)	−0.1917 (3)	0.9503 (2)	0.0202 (7)	
H10B	0.8992	−0.1998	0.9607	0.024*	
H10A	0.6660	−0.1223	1.0054	0.024*	
Cl2	1.15091 (12)	−0.57052 (9)	0.81161 (5)	0.0251 (2)	
Cl1	0.71393 (12)	1.24682 (9)	0.03128 (5)	0.0240 (2)	

### Atomic displacement parameters (Å^2^)

**Table:**

	*U*^11^	*U*^22^	*U*^33^	*U*^12^	*U*^13^	*U*^23^
O1	0.0512 (17)	0.0260 (12)	0.0199 (11)	−0.0198 (12)	−0.0059 (10)	−0.0041 (9)
O2	0.0408 (15)	0.0195 (11)	0.0184 (11)	−0.0124 (11)	−0.0042 (10)	−0.0036 (9)
N1	0.0291 (17)	0.0326 (15)	0.0148 (13)	−0.0124 (13)	−0.0004 (11)	−0.0010 (11)
N2	0.0277 (16)	0.0206 (14)	0.0224 (14)	−0.0095 (13)	−0.0025 (12)	−0.0060 (11)
N3	0.0330 (17)	0.0177 (13)	0.0162 (12)	−0.0125 (12)	0.0004 (11)	−0.0027 (10)
N4	0.0304 (16)	0.0170 (13)	0.0163 (12)	−0.0106 (12)	−0.0030 (11)	−0.0030 (10)
N5	0.0267 (16)	0.0202 (13)	0.0191 (13)	−0.0084 (12)	−0.0010 (11)	−0.0010 (10)
C1	0.0218 (19)	0.0225 (17)	0.0353 (19)	−0.0076 (15)	−0.0070 (15)	0.0070 (15)
C2	0.0193 (18)	0.0114 (15)	0.0254 (16)	−0.0051 (13)	−0.0008 (13)	−0.0032 (12)
C3	0.0227 (19)	0.0222 (17)	0.0223 (16)	−0.0085 (15)	−0.0035 (14)	−0.0018 (13)
C4	0.0229 (19)	0.0242 (16)	0.0186 (15)	−0.0093 (15)	−0.0029 (13)	−0.0009 (13)
C5	0.0232 (18)	0.0178 (15)	0.0180 (15)	−0.0053 (14)	−0.0033 (13)	−0.0019 (12)
C6	0.0210 (19)	0.0240 (17)	0.0202 (16)	−0.0080 (15)	−0.0023 (13)	−0.0023 (13)
C7	0.0255 (19)	0.0165 (15)	0.0151 (15)	−0.0070 (14)	−0.0011 (13)	−0.0008 (12)
C8	0.0228 (19)	0.0217 (16)	0.0186 (15)	−0.0100 (14)	−0.0031 (13)	−0.0026 (12)
C9	0.0128 (17)	0.0208 (16)	0.0230 (16)	−0.0062 (14)	0.0002 (13)	−0.0035 (13)
C10	0.0221 (18)	0.0154 (15)	0.0234 (16)	−0.0055 (14)	−0.0045 (14)	−0.0033 (12)
Cl2	0.0295 (5)	0.0243 (4)	0.0217 (4)	−0.0097 (4)	−0.0006 (3)	−0.0055 (3)
Cl1	0.0291 (5)	0.0241 (4)	0.0215 (4)	−0.0114 (4)	−0.0050 (3)	−0.0022 (3)

### Geometric parameters (Å, °)

**Table:**

O1—C6	1.235 (3)	C1—H1	0.9500
O2—C9	1.237 (3)	C2—C3	1.356 (4)
N1—C1	1.311 (4)	C2—C4	1.495 (4)
N1—C3	1.378 (3)	C3—H3	0.9500
N1—H1'	0.8800	C4—C5	1.514 (4)
N2—C1	1.322 (4)	C4—H4B	0.9900
N2—C2	1.384 (3)	C4—H4A	0.9900
N2—H2'	0.8800	C5—H5B	0.9900
N3—C6	1.333 (3)	C5—H5A	0.9900
N3—C5	1.454 (3)	C6—C7	1.510 (4)
N3—H3'	0.8800	C7—C8	1.515 (3)
N4—C9	1.332 (3)	C7—H7B	0.9900
N4—C8	1.453 (3)	C7—H7A	0.9900
N4—H4'	0.8800	C8—H8B	0.9900
N5—C10	1.483 (3)	C8—H8A	0.9900
N5—H5C'	0.9100	C9—C10	1.510 (4)
N5—H5B'	0.9100	C10—H10B	0.9900
N5—H5A'	0.9100	C10—H10A	0.9900
			
C1—N1—C3	109.9 (2)	H4B—C4—H4A	107.9
C1—N1—H1'	125.0	N3—C5—C4	109.9 (2)
C3—N1—H1'	125.0	N3—C5—H5B	109.7
C1—N2—C2	109.1 (2)	C4—C5—H5B	109.7
C1—N2—H2'	125.4	N3—C5—H5A	109.7
C2—N2—H2'	125.4	C4—C5—H5A	109.7
C6—N3—C5	120.2 (2)	H5B—C5—H5A	108.2
C6—N3—H3'	119.9	O1—C6—N3	120.1 (3)
C5—N3—H3'	119.9	O1—C6—C7	122.2 (2)
C9—N4—C8	121.1 (2)	N3—C6—C7	117.8 (2)
C9—N4—H4'	119.4	C6—C7—C8	110.2 (2)
C8—N4—H4'	119.4	C6—C7—H7B	109.6
C10—N5—H5C'	109.5	C8—C7—H7B	109.6
C10—N5—H5B'	109.5	C6—C7—H7A	109.6
H5C'—N5—H5B'	109.5	C8—C7—H7A	109.6
C10—N5—H5A'	109.5	H7B—C7—H7A	108.1
H5C'—N5—H5A'	109.5	N4—C8—C7	110.9 (2)
H5B'—N5—H5A'	109.5	N4—C8—H8B	109.5
N1—C1—N2	108.3 (2)	C7—C8—H8B	109.5
N1—C1—H1	125.9	N4—C8—H8A	109.5
N2—C1—H1	125.9	C7—C8—H8A	109.5
C3—C2—N2	106.4 (2)	H8B—C8—H8A	108.0
C3—C2—C4	132.2 (2)	O2—C9—N4	123.5 (2)
N2—C2—C4	121.4 (2)	O2—C9—C10	121.6 (2)
C2—C3—N1	106.3 (2)	N4—C9—C10	114.9 (2)
C2—C3—H3	126.9	N5—C10—C9	110.0 (2)
N1—C3—H3	126.9	N5—C10—H10B	109.7
C2—C4—C5	111.9 (2)	C9—C10—H10B	109.7
C2—C4—H4B	109.2	N5—C10—H10A	109.7
C5—C4—H4B	109.2	C9—C10—H10A	109.7
C2—C4—H4A	109.2	H10B—C10—H10A	108.2
C5—C4—H4A	109.2		

### Hydrogen-bond geometry (Å, °)

**Table:**

*D*—H···*A*	*D*—H	H···*A*	*D*···*A*	*D*—H···*A*
N1—H1'···Cl1	0.88	2.27	3.083 (4)	153
N2—H2'···O1^i^	0.88	1.81	2.670 (4)	165
N3—H3'···O2^i^	0.88	2.07	2.927 (4)	165
N4—H4'···Cl1^ii^	0.88	2.31	3.192 (4)	178
N5—H5C'···Cl2^iii^	0.91	2.32	3.152 (4)	152
N5—H5B'···Cl2	0.91	2.32	3.191 (4)	160
N5—H5A'···Cl1^iv^	0.91	2.31	3.161 (4)	156

Symmetry codes: (i) *x*, *y*+1, *z*; (ii) *x*, *y*−1, *z*+1; (iii) −*x*+2, −*y*−1, −*z*+2; (iv) −*x*+1, −*y*+1, −*z*+1.
